# Sometimes the apple does fall far from the tree: a case study on automatic indexing precision errors in PubMed

**DOI:** 10.5195/jmla.2025.2110

**Published:** 2025-10-23

**Authors:** Paije Wilson

**Affiliations:** 1 paije.wilson@wisc.edu, Health Sciences Librarian, University of Wisconsin-Madison School of Medicine and Public Health, Madison, WI

**Keywords:** Abstract and Indexing, MEDLINE, Medical subject heading, automatic indexing, PubMed

## Abstract

**Objective:**

This case study identifies the presence and prevalence of precision indexing errors in a subset of automatically indexed MEDLINE records in PubMed (specifically, all MEDLINE records automatically indexed with the MeSH term Malus, the genus name for apple trees). In short, how well does automatic indexing compare [figurative] apples to [literal] apples?

**Methods:**

1,705 MEDLINE records automatically indexed with the MeSH term *Malus* underwent title/abstract and full text screening to determine whether they were correctly indexed (i.e., the records were about *Malus*, meaning they discussed the literal fruit or tree) or incorrectly indexed (i.e., they were not about *Malus*, meaning they did not discuss the literal fruit or tree). The context and type of indexing error were documented for each erroneously indexed record.

**Results:**

135 (7.9%) records were incorrectly indexed with the MeSH term *Malus*. The most common indexing error was due to the word “apple” being used in similes, metaphors, and idioms (80, or 59.2%), with the next most common error being due to “apple” being present in a name or term (50, or 37%). Additional indexing errors were attributed to the use of “apple” in acronyms, and, in one case, a reference to Sir Isaac Newton.

**Conclusion:**

As indicated by this study's findings, automatic indexing can commit errors when indexing records that have words with non-literal or alternative meanings in their titles or abstracts. Librarians should be mindful of the existence of automatic indexing errors, and instruct authors on how best to ameliorate the effects of them within their own manuscripts.

## INTRODUCTION

MEDLINE is a selective, massive, and ever-growing bibliographic database of primarily biomedical citations [1, 2]. As of 2024, MEDLINE had over 32 million citations, with over 912 thousand references having been added in the year 2024, alone [1]. A common method of searching MEDLINE is by using the PubMed database, an openly available database of biomedical literature which includes all MEDLINE citations, PubMed Central, and NCBI's Bookshelf [3]. One means of facilitating the discovery of MEDLINE citations in PubMed is the application of Medical Subject Headings (MeSH). Created in 1954 by the National Library of Medicine (NLM), MeSH is controlled vocabulary used to index, catalog, and search for biomedical and health-related information in NLM databases [4, 5]. Within the context of PubMed, MeSH terms are exclusively applied to MEDLINE citations [6].

MeSH terms help searchers to at least partly ameliorate the effects of alternative phrasings for a concept [5]. For example, if a MEDLINE record discusses “heart attacks” it may be indexed with the MeSH term, *myocardial infarction*. So long as this MeSH term is assigned to the citation, a search of *Myocardial infarction* as a MeSH term should retrieve this citation, even if the citation only ever mentions “heart attacks” and not “myocardial infarction” in the title or abstract. In addition to facilitating citation retrieval, MeSH terms have also been shown to improve the precision of searches in PubMed as compared to text word searching [7–9]. Traditionally, indexing (i.e., in this context, assigning MeSH terms to MEDLINE citations) entailed indexers reviewing citations and their full text and assigning MeSH terms that best reflected the topics represented in the record. [10–12]. While semi-automation had been introduced to the process in 2002, where indexers were given optional indexing suggestions by the Medical Text Indexer (MTI) algorithm [10, 13–16], indexing was for the most part done manually by indexers at the NLM up until 2011 [17]. As MEDLINE grew, however, the practice of manual indexing became unsustainable, both from workload and financial standpoints, and so the NLM began exploring methods of fully automating the indexing process using algorithms [10–12, 18]. In 2011, NLM experimented with first line indexing using MTI on a selection of 14 journals, wherein MTI automatically assigned MeSH terms to citations from these journals, which were later reviewed by human indexers [10, 14, 16, 17]. Full automated indexing with subsequent versions of MTI-Auto (a.k.a. MTIA, an updated version of MTI) was later applied to citations in OLDMEDLINE in 2015, comments in 2016, and batches of backlogged citations in 2016 [14]. Beginning in April of 2022, fully automated indexing was implemented for all MEDLINE journals using a version of MTIA [17], with a new, machine-learning-based algorithm called Medical Text Indexer-NeXt generation (MTIX) replacing MTIA in 2024 [17, 19]. The precise mechanisms of these algorithms are complex; to simplify, MTI and MTIA algorithms have for the most part relied on keyword frequencies in the title and abstract, keyword locations (e.g., whether the keyword occurred in the title or the abstract, with title receiving greater relevancy ranking), and indexing of PubMed related citations (i.e., MEDLINE records that have similar keywords in their titles or abstracts) to generate their outputs, with a multitude of refinements via a series of rules [10, 14, 16, 17, 19, 20]. MTIX is the first machine learning model of the algorithm, which allows the algorithm to be trained on previously indexed records (specifically the records' title, abstract, publication year, indexing year, and journal name), and, from these data, assign statistically likely MeSH terms to new MEDLINE records added to PubMed [17, 19, 20]. Due to licensing restrictions, none of the existing algorithms have analyzed the full text of MEDLINE articles [10, 17, 19].

The implementation of fully automated indexing has dramatically improved indexing efficiency. Previously, manual indexing had taken a month or more for a single citation (some studies have even shown manual indexing taking several months [21–23]!). With automatic indexing, however, citations can be indexed within a single day [17, 19].

While automatic indexing has been shown to improve efficiency, there have been concerns relating to its accuracy. Many of these concerns stem from the fact that automatic indexing algorithms in PubMed are for the most part limited to only assessing the titles and abstracts of records (unlike manual indexing, which had involved indexers assessing full text records), which can result in the algorithms missing context in the full text that may be absent in the title or abstract of the record [12, 17, 19]. Indeed, reviews have been mixed with regards to automatic indexing's effects on precision and recall (i.e., the relevance and comprehensiveness of the MeSH terms automatic indexing assigns to citations), with some concerning observations including automatic indexing's variable performance between journals and subjects [13, 18, 24, 25], exclusion of relevant MeSH terms [13, 24], and assignment of irrelevant MeSH terms [13, 26].

Most published studies have focused on errors in recall. Chen et al.' s (2023) study of the 2011 version of MTI found that citations from journals from allied health or more specialized domains received fewer MeSH terms from MTI than those from journals from more general or popular biomedical fields, and additionally found that terms associated with non-medical or allied health topics received lower relevancy rankings [13]. Similarly, Llimos et al's (2024) study found that citations from pharmacy practice journals had fewer MeSH terms assigned to them by MTI than those from general medicine journals, and were missing relevant MeSH terms [24]. The findings from Chen and Llimos' studies are concerning, as reducing the number of MeSH terms assigned to a citation can have negative repercussions on the citation's retrievability in PubMed.

A few studies have evaluated the precision of different versions of MTI. Mork et al's (2017) study found that NLM indexers reacted positively to MTI's suggestions, with usage of MTI's suggestions by NLM indexers increasing from 15.75% in 2002 to 62.44% in 2014; and that MTI's precision had steadily improved from .3019 in 2007 to between .6003 and .64 in 2014 [10]. Moore et al.'s (2024) study reported a 53% precision for grants, 73% for patents, and 64% for drug indications [26].

While the aforementioned studies have evaluated precision in the general sense, very few studies have identified specific precision errors, with such errors often being remarked upon in passing, rather than systematically documented. Such precision errors have included MTI misinterpreting counterindications in drug indications text [26] and, in one case, assigning a MeSH term that didn't represent the subject of the citation [13]. There have also been a number of anecdotal observations of these indexing errors, including in a webinar hosted by the NLM, which mentioned MTI's poor performance with metaphors [27]; and in a few librarians' social media and listserv posts, which have pointed out various (and sometimes comical) indexing errors for citations containing words with non-literal or alternative meanings [28]. Such errors can have dramatic impacts on the retrieval of records in PubMed, as incorrect indexing may not only introduce clutter to the results of searches for systematic evidence syntheses, but, in cases where correct index terms are absent, may negatively impact records' discoverability. Despite these risks, few, if any, studies have given special focus on identifying the presence and prevalence of precision errors in records automatically indexed in PubMed.

To fill this gap, this case study investigates whether automated indexing can appropriately and precisely apply MeSH terms in the context of non-literal or alternative meanings. The MeSH term *Malus* (being the genus name for apple tree) was chosen due to the common use of the word “apple” in figurative contexts (e.g., “comparing apples to oranges,” “apple of one's eye,”), its capacity to have alternative meanings (e.g., Apple, Inc., apple snails), and the manageable number of citations in the sample.

## METHODS

There are three ways by which MEDLINE citations can be indexed in PubMed: manual (which refers to citations that were indexed solely by human indexers), curated (which refers to citations that were indexed automatically, then were later reviewed by human indexers), and automated (which refers to citations that were automatically indexed, and did not undergo review by human indexers) [29]. The indexing method of a citation in PubMed has been recorded into the XML files of citations since 2018 (with “curated” and “automated” labels being assigned to curated and automatically indexed citations, respectively, and the absence of a label indicating manually indexed citations) [29].

Automatically indexed citations (i.e., citations that were automatically indexed and did *not* undergo review by human indexers) can be retrieved in PubMed by applying the following string to a search strategy: indexingmethod_automated [17]. With this in mind, a search was constructed to retrieve all citations automatically indexed with the MeSH term, *Malus*. The search strategy is provided below. No additional filters were applied to the search.

### Malus[mesh] AND indexingmethod_automated

The search was run on June 26, 2024, with all results being exported from PubMed as an .nbib file and imported into EndNote 21. Title/abstract screening was conducted for individual records in EndNote 21 using the summary tab of the preview pane, during which time records were categorized as being correctly indexed (i.e., they were about *Malus*, meaning they discussed the literal fruit or tree), incorrectly indexed (i.e., they were not about *Malus*), or uncertain (i.e., the reviewer was not sure whether the record was correctly or incorrectly indexed). Each reference was assigned an EndNote 21 tag corresponding with its category (i.e., correct indexing, incorrect indexing, or uncertain). The records were then exported into an Excel sheet.

Records identified as incorrectly indexed or uncertain then underwent full text screening in Excel, during which time the reviewer identified records as being correctly indexed, incorrectly indexed, or where the classification could not be determined (i.e., the full text couldn't be accessed to verify whether the record was correctly or incorrectly indexed).

Records identified as being incorrectly indexed during full text screening then underwent data extraction, during which time the reviewer copied a quotation of the context in which variations of the word “apple” or “*Malus*” were used in the record. These quotations were pasted into the Excel sheet, and, in a separate column, were assigned into categories in accordance with their context (e.g., simile, metaphor, acronym, etc.).

To gain some insight into the performance of NLM's new MTIX algorithm, citations indexed using MTIX were identified and labelled in the Excel sheet. According to the NLM HelpDesk, MTIX was officially implemented in PubMed on 4/23/2024 [30]. With this in mind, to identify citations that were automatically indexed using the MTIX algorithm, a search was run of all the citations' PMIDs from the Excel sheet (regardless of whether they were labeled as being correctly or incorrectly indexed, or if the correctness of indexing could not be determined) combined with a date indexed filter, with the filter starting on 4/23/2024 and ending in the year 3000, using the [mhda] field tag in PubMed. An abridged version of this strategy with only 3 PMIDs is provided below. Records retrieved by this search were labeled as having been indexed by MTIX in the Excel sheet.

**(23862187 OR 38729358 OR 38363483) AND (2024/04/23:3000[mhda])**

## RESULTS

**Figure 1:**
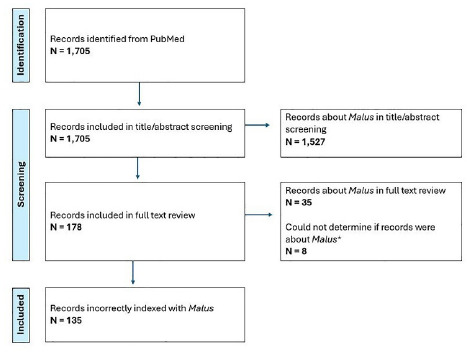
Flow diagram of MEDLINE records automatically indexed with the MeSH term, *Malus*. Flow diagram adapted from the PRISMA Flow Diagram from Page et al. (2021) [31].

**Figure 2:**
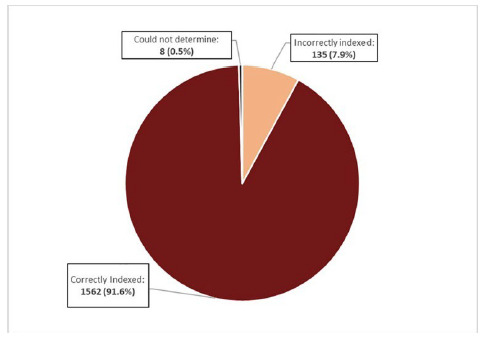
Pie chart of MEDLINE records automatically indexed with the MeSH term, *Malus*. The categories denote whether the records were indexed correctly, indexed incorrectly, or where the correctness of the indexing could not be verified.

The search retrieved a total of 1,705 records, with just 82 of these records being indexed by MTIX. During title/abstract screening, 1,527 records were identified as being correctly indexed, and were excluded from full text screening. The remaining 178 records then underwent full text screening, during which time 35 records were identified as being correctly indexed, and 8 records were inconclusive (i.e., the full text could not be accessed to verify whether they were correctly or incorrectly indexed). This left 135 records that were identified as incorrectly indexed. In sum, of the 1,705 records retrieved, 1,562 (91.6%) were correctly indexed, 135 (7.9%) were incorrectly indexed, and 8 (0.5%) were inconclusive (see **Figures 1** and **2**).

*8 records' full text could not be accessed. They were therefore labeled as “could not determine.”

**Figure 3:**
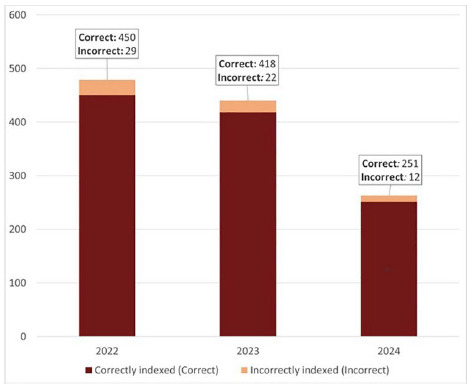
Bar chart of MEDLINE records with publication years between 2022 and 2024 automatically indexed with the MeSH term *Malus* that were indexed correctly, indexed incorrectly, or where the correctness of the indexing could not be verified.

The majority of records automatically indexed with the MeSH term, *Malus,* were published between 2020 and 2024 (1,475, or 87%) (see **Table 1**). Of these 1,475 records, 94 (6%) were incorrectly indexed. When isolating data from 2022, 2023, and 2024, the percentage of incorrectly indexed records remained fairly consistent, being at 6% (29 of the 479 records), 5% (22 of the 440 records), and 5% (12 of the 263 records), respectively (see **Figure 3**). All 82 MTIX indexed records indexed with the MeSH term *Malus* were correctly indexed.

**Table 1:** Table of MEDLINE records automatically indexed with the MeSH term *Malu*s by publication year that were indexed correctly, indexed incorrectly, or where the correctness of the indexing could not be verified. A time lapse is present between 1970 and 2004, during which time no records were automatically indexed with the MeSH term *Malus.*

Years	Correctly indexed	Incorrectly indexed	Could not determine	Totals
1945 - 1949	30	2	1	33
1950 - 1954	46	1	2	49
1955 - 1959	37	1	1	39
1960 - 1964	27	1	3	31
1965 - 1969	8	0	0	8
2005 - 2009	1	0	0	1
2010 - 2014	4	1	1	6
2015 - 2019	28	35	0	63
2020 - 2024	1381	94	0	1475

Interestingly, there were some records published prior to 2022 that were automatically indexed (230, or 13% of the 1,705 records). As the NLM HelpDesk confirmed that MEDLINE citations are not automatically indexed retrospectively [32], these older records are likely attributed to automatic indexing being applied since 2015 to citations in OLDMEDLINE (which includes citations published between 1946 through 1965 [33]), and since 2016 for comments and batches of backlogged citations (the latter of which may have included some citations with publishing dates prior to 2016) [14].

Of the 135 records incorrectly indexed with the MeSH term *Malus* the most common automated indexing error was misinterpreting metaphors, similes, and idioms (80, or 59%). These included variations of phrases such as “like comparing apples to apples”, “apples falling far from the tree”, “bad apples”, “an apple a day keeps the doctor away”, and “apple of one's eye.” They also included references to things resembling apples (i.e., “apple-shaped” body types and “lymphoid hyperplasia resembling apple tree branches”).

Another automated indexing error observed in this sample included references to names or terms that included “apple” (being 50 records, or 37% of the 135 records). Specifically, this included references to the names Apple, Inc. (23, or 17% of the 135 records); the Miyake-Apple Technique (being a photographic/video analysis technique for cataract surgery [34]) (8, or 6% of the 135 records); plants with “apple” in the term that were not from the genus *Malus* (e.g., thorn apples) (7, or 5% of the 135 records); the Apple Domain (being in reference to amino acid domains) (4, or 3% of the 135 records); apple peel jejunal atresia (being a form of jejuna atresia [35]) (4, or 3% of the 135 records); apple snails (2, or 1% of the 135 records); “the Big Apple” (being a reference to New York) (1, or 1% of the 135 records); and apple bite fractures (being a type of fracture in the posterolateral tibia plateau [36]) (1, or 1% of the 135 records).

**Figure 4:**
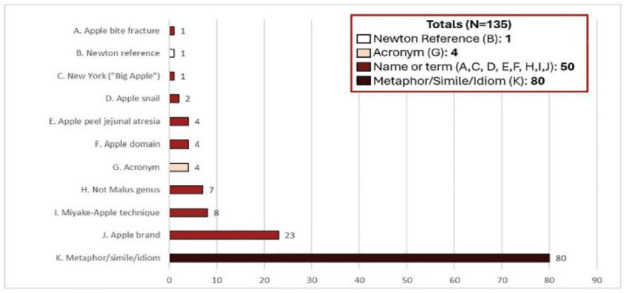
Bar chart of indexing errors in records automatically indexed incorrectly with the MeSH term, *Malus*.

There were also automated indexing errors when records used acronyms (e.g., “Access to Post Partum LARC in Edinburgh South (APPLES)”) (4, or 3% of the 135 records), and, in a single instance (1% of the 135 records), a passing reference to Sir Isaac Newton (see **Figure 4**).

## DISCUSSION

This case study, although narrow in focus, shows that PubMed's automated indexing algorithms do make errors when indexing records that include words with non-literal or alternative meanings. Within the context of records automatically indexed with the MeSH term *Malus*, these errors comprised approximately 8% of this study's sample, with the majority of errors stemming from the use of the word “apple” in metaphors, similes, and idioms. Additional errors that were noticed were when a variation of the word “apple” was used for names or terms, acronyms, and, in one instance, a reference to Sir Isaac Newton. With approximately 8% of just this limited sample containing automatic indexing errors, and what with the passing observations from previous studies and anecdotes, it's arguably safe to assume citations containing other words with non-literal or alternative meanings may have similar indexing errors. This leads one to wonder at the prevalence of such errors in broader fields, such as public health or nursing. Such errors negatively affect the precision of a search, being one of the very obstacles, ironically, that MeSH had been designed to ameliorate [5, 7].

In addition to increasing the number of irrelevant records to screen in evidence syntheses (which can already be a burdensome feat, a fact of which many librarians are all too aware), these errors can also reduce the retrievability of relevant records. For example, take an editorial in *Nature Cancer* entitled “Tackling Metastasis” [37]. As of May 20, 2025, the record has been erroneously assigned the MeSH terms, *Football* and *Athletic Performance* with no MeSH terms listed relating to cancer. Not only would such a record add to the clutter retrieved by a search relating to football, but it would also fail to be retrieved if one were to rely on using cancer-related MeSH terms in their search.

The impact of automatic indexing errors will be particularly hard-felt by subjects that have less optimal indexing such as allied health, pharmacy practice, and non-medical journals [13, 24]. If a record from a subject that receives fewer MeSH terms happens to be assigned an incorrect MeSH term by automatic indexing, it will be less likely to have additional, relevant MeSH terms to counterbalance the effects of the erroneous one, as compared to records from more popular biomedical fields. For example, a record about falling risks after fractures was incorrectly indexed (as of May 21, 2025) with the MeSH term *Seasons* [38], but the record has additional, relevant MeSH terms, such as *Aged* and *Fractures, Bone* which may result in the record still being retrieved by a sensitive search about falls. However, our aforementioned “Tackling Metastasis” one does not have any additional, relevant MeSH terms relating to cancer to increase its likelihood of being retrieved by a cancer-specific MeSH search. Automatic indexing's variable recall performance between subjects, paired with the precision errors noted in this study, will only further perpetuate biases within PubMed, as citations from well-indexed subjects will be more likely to be retrieved in PubMed than those from subjects whose indexing is less optimal [13, 24]. Apart from including potential, erroneously-assigned MeSH terms in their searches, and risking the retrieval of even more clutter in their search results, librarians may be forced to rely on keyword searching (i.e., searching in the titles or abstracts) to retrieve such records.

So, what can be done to address precision errors in PubMed's automatic indexing? One means of addressing these errors is to simply bring more attention to them. This can be done by conducting additional, systematic, and larger-scale studies on common automatic indexing errors, being especially important with the recent implementation of MTIX, NLM's newest automated indexing algorithm, in 2024 [19]. While this study did look at a subset of MTIX indexed records (82 total), with, promisingly, all of these records being correctly indexed with the MeSH term *Malus*, a larger sample would be needed to effectively evaluate MTIX's precision. Future studies can additionally focus on the performance of MTIX over time (as this study was conducted less than a year after MTIX's implementation), and whether MTIX missed relevant MeSH terms when indexing records (which was outside the scope of this study). Less formally, PubMed users can report indexing errors they encounter via the NLM HelpDesk [17, 39]. As MTIX operates using a form of artificial intelligence called a neural network, which allows it to use past training data to “learn” how to index new records [19], it's possible that reporting indexing errors may help NLM researchers to further train and refine the algorithm.

Some studies have argued that automatic indexing may be improved if the MTIX algorithm is given access to the full text of records in PubMed [17, 19]. Unlike manual indexing, which had analyzed the full text of records, MTIX is currently limited to analyzing the titles and abstracts of records [12, 17, 19]. In consequence, the algorithm may miss much needed context that may be present in the full text but which is absent in the title or abstract. There have been concerns relating to the use of full text for the purposes of retrieval in databases, including scalability (as full text documents are considerably longer than abstracts, and will therefore require more effort for algorithms to process), variable file types (which can complicate processing), variations in the structure of the article (e.g., labeling “methods” as “methodology” can compromise an algorithm's ability to pinpoint specific sections of text for analysis), and the potential for long texts to negatively impact precision (as more text could amount to more “noise” picked up by an algorithm) [40–42]. In fact, past studies examining indexing using blocks of full text have found that the inclusion of full text can have negative effects on indexing precision [43, 44]. However, there have been noted improvements to automated indexing when full text is processed in sections (e.g., introduction, methods, results, etc.), rather than as intact blocks of text. Both Dai et al.'s (2020) and You et al.'s (2021) studies found that indexing algorithms trained on sectioned full text from PubMed Central (PMC) significantly improved indexing performance as compared to indexing algorithms that relied solely on title and abstract processing (such as MTI) [41, 42]. Similarly, Lin's (2009) study on full text searching in the TREC 2007 genomics track evaluation data found that segmented full text searching added significant value to retrieval as compared to title and abstract searching, alone [40]. While current licensing restrictions prohibit PubMed's indexing algorithms from accessing the full text, the NLM has reported they are investigating the possibility of this option in the future [17].

Finally, librarians can present authors with workarounds to help them mitigate these kinds of indexing errors in their own records. Librarians can do this by advising authors to use more descriptive, and standardized terminology in the titles and abstracts of their manuscripts; for example, by using the very MeSH terms they would like to see assigned to their record [9, 13, 17, 24, 45] and avoiding the use of non-literal language, such as metaphors and similes, in the title and abstract whenever possible [13, 27]. While the use of words with non-literal or alternative meanings cannot be wholly avoided in the title and abstract, the use of descriptive, and standardized terminologies can at least increase the likelihood of correct MeSH terms being applied to the record alongside the erroneous ones. As librarians provide support for researchers in database searching and frequently assist in the publication process, they are in the optimal position to advise authors on the limitations of automatic indexing and provide tips on how to ensure their manuscripts are more discoverable.

## LIMITATIONS

While this case study provides insight on precision errors in automatic indexing, the sample was extremely limited (being limited to only records indexed under the MeSH term *Malus*). Future studies can be conducted to examine larger samples of specific indexing errors, especially within the context of words with non-literal or alternative meanings. Studies examining precision are especially needed with the recent implementation of MTIX in 2024, as they could provide insight into the new algorithm's performance over time. This study was additionally limited to examining precision errors in automatic indexing, and not recall (i.e., the exclusion of relevant MeSH terms), being a type of error that can have significant impacts on retrieval. Additional studies focusing on recall, especially within the context of MTIX, are warranted. Future studies can also compare the precision of automated indexing with curated and manual indexing.

## CONCLUSIONS

While limited, this case study provides insight into specific precision errors in automatic indexing for MEDLINE records in PubMed. As indicated by this study's findings, automatic indexing generates errors when it encounters records that have words with non-literal or alternative meetings in their titles or abstracts, such as names or terms, similes, metaphors, acronyms, and idioms. If precision errors were noticed in such a limited sample, one wonders at the prevalence of such errors in broader disciplines, such as nursing or public health. While a few “rotten apples” (i.e., precision errors) may not ruin the “batch” (i.e., search functionality in PubMed), compounding precision errors can decrease the utility of MeSH indexing and compromise the discoverability of MEDLINE records in PubMed, especially records deriving from fields with less optimal indexing. Studies such as this (especially at a larger scale) can bring attention to these errors, and inform future modifications to PubMed's automatic indexing algorithm. In the meantime, librarians should be mindful of the existence of automatic indexing errors, and advise future authors on how best to ameliorate their effects within their own manuscripts. Perhaps, through these means, we can kick the apples just a little bit closer to their trees.

## Data Availability

All data used for this study can be accessed at https://github.com/weepai/Sometimes-the-apple-does-fall-far-from-the-tree
