# Supplementary material for: Sometimes the apple does fall far from the tree: a case study on automatic indexing precision errors in PubMed
Source: J Med Libr Assoc. 2025 Oct 23;113(4):318–26. doi: 10.5195/jmla.2025.2110 (PMC12606386; doi:10.5195/jmla.2025.2110)

# Appendix 1

**Figure:** Bar chart of subset of records published between 2022 and 2024 automatically indexed correctly and incorrectly with the MeSH term, *Malus*, by publication year.


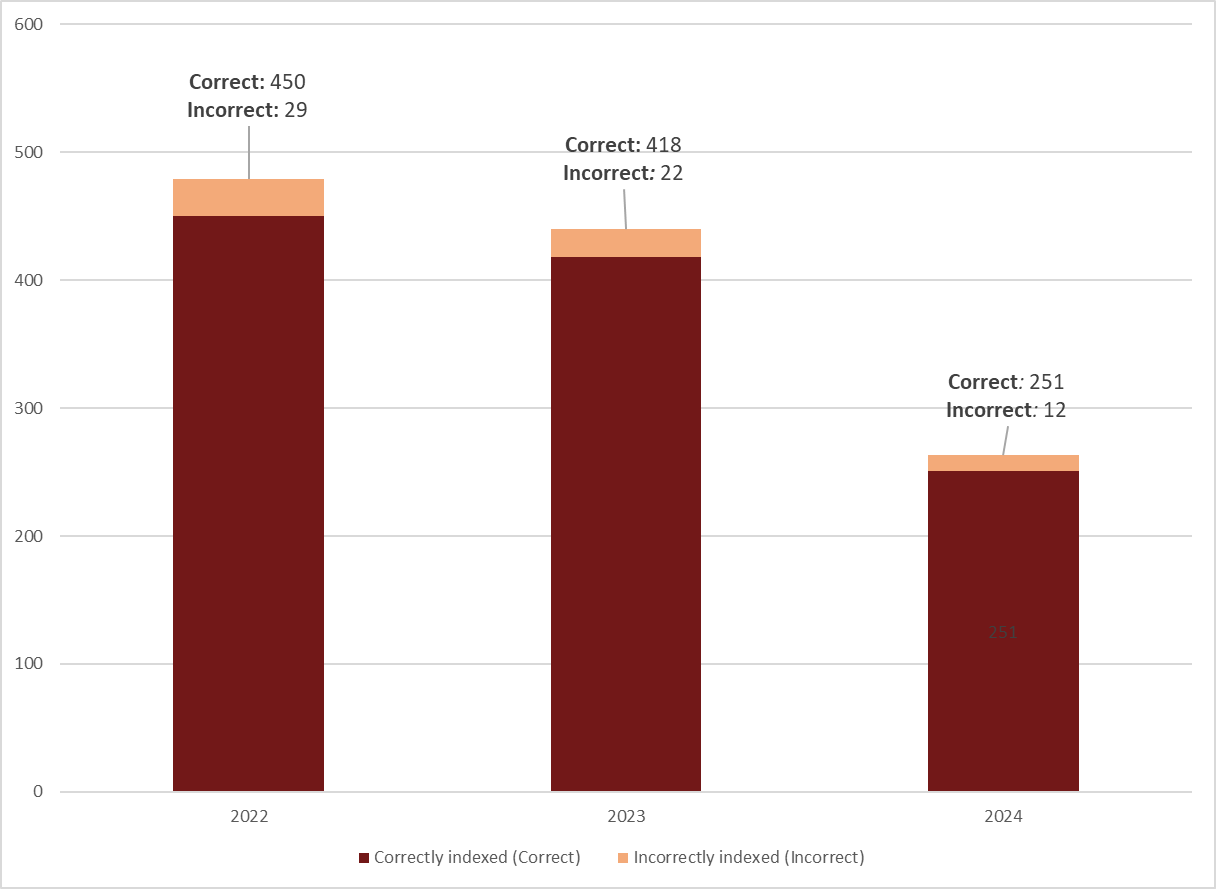

Supplement: Supplementary file 1 — Appendix A [file jmla-113-4-318-s01.docx]
